# Orthopaedic Program Reputation and Association With Social Media Utilization and Engagement

**DOI:** 10.5435/JAAOSGlobal-D-22-00095

**Published:** 2022-11-09

**Authors:** Peter Y. Joo, Jay Moran, Christopher Wilhelm, John Ready, Lee E. Rubin, Jonathan N. Grauer

**Affiliations:** From the Department of Orthopaedics and Rehabilitation Yale School of Medicine, New Haven, CT.

## Abstract

The associations of orthopaedic social media metrics with *US News & World Report* (USNWR) scores have not been well defined and are the focus of this study.

**Methods:**

Orthopaedic surgery departments and residency programs were matched to the USNWR overall orthopaedic score and professional opinion subscore. Corresponding Instagram and Twitter accounts were evaluated for the number of followers, number following, and posts. Correlations between these metrics and rank/reputation were assessed. Pearson correlations and Student *t* tests were used with significance set at *P* < 0.05.

**Results:**

Of the 192 departments associated with residency programs, there were social media accounts for 150 (78.1%) and USNWR rankings for 186 (96.9%), with an overlap of 147 (76.6%). Instagram accounts were identified for 138 (93.9%) and Twitter accounts for 85 (57.8%). Correlations were highest for the opinion subscore and number of followers (Instagram department *R* = 0.894, Instagram residency *R* = 0.338, Twitter department *R* = 0.808, and Twitter residency *R* = 0.878, *P* < 0.001 for each), less for the number of posts (Instagram department *R* = 0.590, Twitter department *R* = 0.521, and Twitter residency *R* = 0.696, *P* < 0.001 for each).

**Conclusion:**

Social media metrics correlated with USNWR scores. Focusing on such social media platforms may help further the reputation, audience engagement, and ranking of orthopaedic departments and residencies.

Social media use has dramatically increased globally, and its use in the healthcare setting has followed this trend.^1–3^ Social media allows users to generate, share, receive, comment, communicate, and connect with others easily and efficiently.^2^ In addition, these platforms facilitate idea sharing, interaction, and discussion.^4^ In the past few years, the social media footprint in graduate medical education and residency recruitment has rapidly expanded.^4^

Regarding orthopaedic social media, several groups are likely to engage with social media: departments, residencies, and individuals. Departments may use social media to engage with the lay public, increase visibility for marketing and advertisement campaigns, and share research or accomplishments of members of the department. Distinct from department social media accounts, orthopaedic residency programs may use social media to engage with applicants and share content related to the culture and social aspects of the program, particularly during the Coronavirus (COVID) pandemic.^5,6^

A potential yardstick with which to assess rank and reputation of orthopaedic programs is the US News & World Report (USNWR) rankings of hospitals, which is based on factors that include patient outcomes, patient experience, nursing staffing, advanced clinical technologies, and professional opinions. Interestingly, the USNWR reports both an overall and professional opinion score for every hospital with orthopaedic services.^7^ These professional opinion scores are calculated from “expert opinions” that are analyzed by the percent of orthopaedic specialists who *perceived* the hospital as the best for very challenging patients.

This study was to examine the utilization of social media by orthopaedic departments and residency programs in the context of associated USNWR overall and professional opinion scores. We hypothesized that orthopaedic surgery departments and residencies with higher USNWR scores would have greater social media presences than those with lower USNWR scores. Such data can provide important information for programs to optimize engagement with prospective residency applicants, professional colleagues, patients, and the general public.

## Methods

This study was considered exempt from the Institutional Review Board because it only uses publicly available data on an organizational level rather than an individual level.

### Accreditation Council for Graduate Medical Education Orthopaedic Programs, Departments, and Social Media Data

All Accreditation Council for Graduate Medical Education (ACGME)–accredited orthopaedic surgery residency programs were identified from the ACGME website.^8^ This list included all allopathic, osteopathic, civilian, and military programs in the United States. Associated departments were then defined.

Publicly available orthopaedic department and associated residency program Instagram and Twitter accounts were identified through a Google search of hospital or residency program name and relevant social media platform. Instagram accounts were searched using the keywords “Department/program name + Instagram,” and Twitter accounts were searched using the keywords “Department/program name + Twitter.” Categorization into “Department” or “Residency Program” was determined based on explicit mention of “Department” or “Residency/Residents” in the account bios.

Instagram and Twitter accounts were analyzed for the number of followers, number of accounts they were following, and number of posts. This information is publicly available on the respective Instagram and Twitter account pages. All data were collected in October 2021. Only those with social media accounts were included in the analyses.

### US News & World Report Rankings

These programs and departments were then matched to the 2021 *US News & World Report* (USNWR) overall orthopaedic rankings which were drawn from 1660 hospitals from across the nation.^7^ The overall orthopaedic ranking is based on analysis of patient outcomes and experiences (49% of score); key programs, services, and staff (20% of score); professional recognition (30.8% of score); and conditions/procedures applicable to orthopaedics such as performance in hip fracture, back surgery, hip arthroplasty, and knee arthroplasty care. For some of the analyses, programs were additionally dichotomized into USNWR “Top 50” programs and “Not Top 50” programs based on overall ranking.

The professional opinion subscores are additionally reported and were additionally abstracted from each institution's USNWR score breakdown on the USNWR website.^7^ These are stated to be based on criteria that include an “expert opinion” score that analyzes the percent of orthopaedic specialists aggregated over the years 2019, 2020, and 2021 who named the hospital as among the best for very challenging patients. This expert opinion percentage was used as the “professional opinion score” for this study.

### Statistical Analysis

Statistical analysis included Pearson correlation analysis of the two USNWR scores stratified by hospitals with and without a Top 50 USNWR ranking, and of each USNWR score with social media followers, following, and posts per department and residency program.

For all correlations, the R and the *P*-values were reported. The correlation coefficient was then compared between the USNWR overall ranking and professional opinion scores of those in the Top 50 and not in the Top 50 using Fisher r-to-z transformation.

Descriptive statistics were extracted and reported using the Statistical Package for the Social Sciences (SPSS; Version 28) software. Significance was set at *P* < 0.05.

## Results

### General

**Figure 1:**
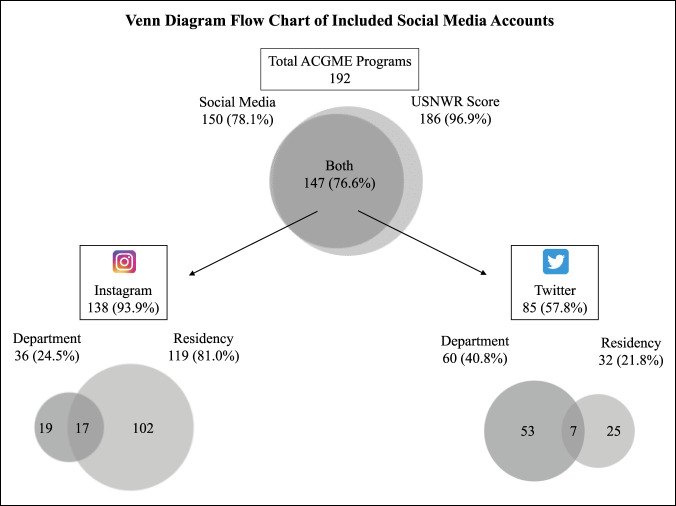
Venn diagrams showing orthopaedic departmental and residency program social media accounts.

A total of 192 ACGME-accredited residency programs and associated departments were identified. Of these, social media accounts were identified for 150/192 (78.1%) and USNWR scores were available for 186 of 192 (96.9%). When considering those with both social media accounts and USNWR rankings, there were 147 of 192 (76.6%) for trend analyses (Figure 1).

Of the 147 institutions included, Instagram accounts were identified for 138 departments and residency programs (93.9%) , with departmental accounts consisting of 36 (24.5%) and residency accounts consisting of 119 (81.0%). Institutions with both departmental and residency accounts were 17 (11.6%).

Twitter accounts were identified for 85 departments and residency programs (57.8%), with departmental accounts consisting of 60 (40.8%) and residency accounts consisting of 32 (21.8%). Institutions with both departmental and residency accounts were 7 (4.8%).

### US News & World Report

The overall USNWR orthopaedic scores ranged from 8.9 to 100.0 (of 100), and the professional opinion subscores ranged from 0.0 to 22.2% (of 100%).

**Figure 2:**
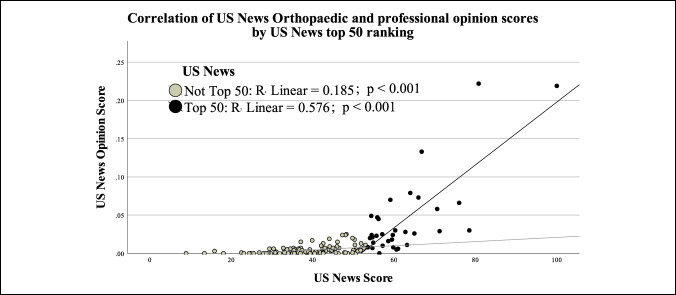
Scatter plot showing correlation between the US News Orthopaedic score and the professional opinion score, with US News Top 50 ranking subgroup analysis showing significant difference in slope between the Top 50 institutions and those not in the Top 50.

The overall USNWR score was then plotted against professional opinion subscores to assess the correlation (Figure 2). Although there were correlations between these variables along the overall USNWR spectrum, the correlation was much greater for those that had scored in the Top 50 category than those in the Not Top 50 category (R linear = 0.576 versus R linear = 0.185, independently both with correlation *P* values < 0.001, comparatively different *P* = 0.010).

### Correlation of US News & World Report Scores and Instagram

The number of Instagram followers for department and residency accounts correlated with the USNWR overall orthopaedic rankings and the professional opinion scores (*P* < 0.001 for each) (Table 1).

**Table 1:** Correlation of US News Overall Orthopaedic Scores and Professional Opinion Scores With Number of Followers, Accounts Following, and Total Social Media Posts of Orthopaedic Social Media Accounts

Factor or Variable	US News Overall Score		Opinion Score	
	Correlation (R)	*P* value	Correlation (R)	*P* value
Instagram				
Followers				
Department	**0.642**	**<0.001**	**0.894**	**<0.001**
Residency	**0.409**	**<0.001**	**0.338**	**<0.001**
Following				
Department	−0.018	0.920	−0.036	0.839
Residency	0.049	0.604	0.023	0.808
Posts				
Department	**0.469**	**0.005**	**0.590**	**<0.001**
Residency	0.113	0.225	0.096	0.299
Twitter				
Followers				
Department	**0.581**	**<0.001**	**0.808**	**<0.001**
Residency	**0.670**	**<0.001**	**0.878**	**<0.001**
Following				
Department	0.227	0.081	0.208	0.110
Residency	0.105	0.572	0.314	0.085
Posts				
Department	**0.427**	**<0.001**	**0.521**	**<0.001**
Residency	**0.446**	**0.012**	**0.696**	**<0.001**

Boldface indicates statistically significant (*P* < 0.05)

The number of Instagram following for department and residency programs did not significantly correlate with either the overall orthopaedic score or the professional opinion score (Table 1). The number of Instagram posts for departmental accounts was significantly correlated with the USNWR overall orthopaedic rankings and the professional opinion scores (*P* = 0.005 and *P* < 0.001); however, this was not true for residency accounts.

### Correlation of US News & World Report Scores and Twitter

The number of Twitter followers for department and residency program accounts correlated with the USNWR overall orthopaedic ranking and the professional opinion score (*P* < 0.001 for each) is summarized in Table 1.

The number of Twitter following for department and residency programs did not significantly correlate with either the overall orthopaedic score or the professional opinion score (Table 1). The number of Twitter posts for departmental and residency accounts was significantly correlated with the USNWR overall orthopaedic rankings and the professional opinion scores (*P* < 0.001 except for residency account and USNWR overall *P* = 0.012).

### Sub Analysis of US News & World Report “Top 50”

**Figure 3:**
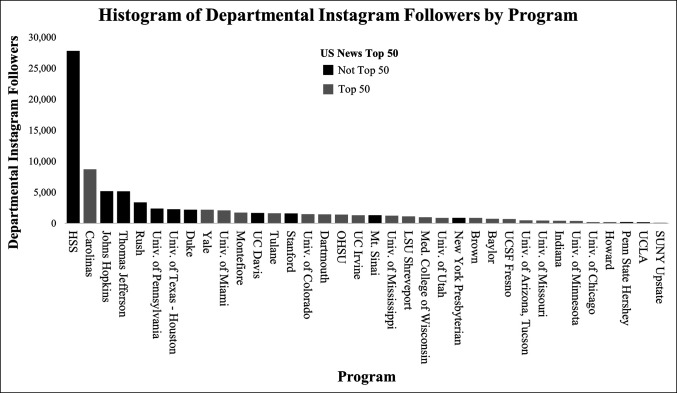
Example histogram showing the group with the highest correlation of the US News Professional Opinion score with the number of social media followers—Instagram accounts of Orthopaedic departments (R = 0.894)—stratified by those in the US News Top 50 ranking and those not in the Top 50 ranking. HSS = Hospital for Special Surgery, LSU = Louisiana State University, OHSU = Oregon Health and Sciences University, SUNY = State University of New York; UC = University of California; UCLA = University of California, Los Angeles; UCSF = University of California, San Francisco.

Departments were then shown in decreasing order of the number of Instagram followers in Figure 3 because this group was shown to be one of the highest correlated with the USNWR scores. This histogram visually depicts orthopaedic departments ranked in the USNWR Top 50 as having higher follower counts, with seven of the top 10 departments (70%) represented as in the USNWR Top 50.

## Discussion

With a rise in social media presence and engagement in the medical community in recent years, there is increasing interest for both orthopaedic surgery departments and residency programs to better understand the utility of social media for marketing, branding, and recruitment. The most important finding in this study was that programs with higher USNWR professional opinion scores were markedly associated with greater social media engagement when compared with programs with lower scores.

These findings confirmed our primary hypothesis and can provide important information for programs to optimize engagement with prospective residency applicants, professional colleagues, patients, and the general public. To the best of our knowledge, this study is the first to analyze correlations between both departmental and residency program social media metrics and USNWR overall orthopaedic and professional opinion scores.

This study found that although the USNWR overall orthopaedic score and professional opinion score were positively correlated, the professional opinion score increased much more for those in the Top 50 versus those not in the Top 50, as has been demonstrated in other areas of marketing.^9^ Similarly, orthopaedic surgeons’ perceptions and opinions of certain institutions may be positively biased by marketing and branding based on the USNWR scores, social media presence, or a combination of both.

Interestingly, residency programs more used Instagram, whereas departments more used Twitter—in line with data from the Pew Research Center that found generational differences in choices of social media platforms.^10^ Bram et al. and Malyavko et al. reported Instagram accounts for 118 and 108 residency programs and Twitter accounts for 38 and 65 residency programs, respectively.^5,6^ As this study differentiated between department-run and residency-run accounts, it is likely that a number of the Twitter accounts that were considered residency program accounts by the previous studies were classified as department-run in this study.

The greatest correlations with USNWR scores were found with social media account followers. This is mostly in line with findings by Triemstra et al. that showed positive correlations for all hospitals/specialties between USNWR reputation scores and Twitter follower count and posts, and positive correlations between USNWR overall rankings and Twitter/Instagram presence.^11^ Similar correlations between ranking and social media followers have been reported for urology and gastrointestinal divisions, highlighting the potential financial implications of social media adoption and utilization.^12,13^ Furthermore, on evaluating differences between those with low and high reputations, social media followers differed markedly, highlighting the association between social media engagement and reputation.

The primary limitation of this study is that cause and effect of the associations observed cannot be determined. In other words, those with greater reputations may be more likely to develop social media presence, as opposed to the opposite. Although the causal link for advertisement campaigns and perception, as well as the magnitude of effect from social media presence, are difficult to track, there exists a significant body of evidence from the private consumer sector on advertisement effects.^1,2,9,14^ Thus, the correlation found in this study may suggest that the social media presence influences perception of certain institutions, although future studies would benefit from prospective tracking to further elucidate temporal relationships. Furthermore, although identification of social media accounts was done in a systematic manner to optimize identification of social media accounts, it is impossible to determine whether every available account was fully captured based on the variations of names and handles that are possible.

## Conclusion

There is increasing interest in social media use, both by orthopaedic surgery departments and residency programs for recruitment and promotion. This study found that orthopaedic departments and residency programs with higher USNWR reputations tended to have larger numbers of followers and greater number of posts for both Instagram and Twitter. The use of social media differs among residency programs and departments with low and high reputations, which may represent opportunities to better leverage social media platforms to optimize engagement with program applicants, professional colleagues, patients, and the general public.

## References

[1] Thackeray R Neiger BL Hanson CL McKenzie JF: Enhancing promotional strategies within social marketing programs: Use of Web 2.0 social media. Health Promot Pract 2008;9:338-343. 18936268 10.1177/1524839908325335

[2] Moorhead SA Hazlett DE Harrison L Carroll JK Irwin A Hoving C: A new dimension of health care: Systematic review of the uses, benefits, and limitations of social media for health communication. J Med Internet Res 2013;15:e85. 23615206 10.2196/jmir.1933 PMC3636326

[3] Boyd DM Ellison NB: Social network sites: Definition, history, and scholarship. J Comput Mediat Commun 2007;13:210-230.

[4] Sterling M Leung P Wright D Bishop TF: The use of social media in graduate medical education: A systematic review. Acad Med 2017;92:1043-1056. 28225466 10.1097/ACM.0000000000001617 PMC5487290

[5] Malyavko A Kim Y Harmon TG : Utility of social media for recruitment by orthopaedic surgery residency programs. JB JS Open Access 2021;6:e21.00076. 10.2106/JBJS.OA.21.00076 PMC8423379 34514283

[6] Bram JT Jia L Huffman W Ahn J: Orthopaedic surgery residency program social media presence during the COVID-19 pandemic. JB JS Open Access 2021;6:e21.00073. 10.2106/JBJS.OA.21.00073 PMC8522873 34671711

[7] United States News and World Report: Best hospitals for orthopedics, 2021, https://health.usnews.com/best-hospitals/rankings/orthopedics. Accessed January 2, 2022.

[8] Accreditation Council for Graduate Medical Education: ACGME accredited program search, https://apps.acgme.org/ads/Public/Programs/Search. Accessed January 2, 2022.

[9] Perkins KA Karelitz JL: Acute perceptions of preferred cigarettes when blinded to brand. Tob Control 2019;28:311-316. 29991640 10.1136/tobaccocontrol-2018-054388

[10] Pew Research Center: Demographics of social media users and adoption in the United States, https://www.pewresearch.org/internet/fact-sheet/social-media/. Accessed December 23, 2021.

[11] Triemstra JD Poeppelman RS Arora VM: Correlations between hospitals' social media presence and reputation score and ranking: Cross-sectional analysis. J Med Internet Res 2018;20:e289. 30409768 10.2196/jmir.9713 PMC6250955

[12] Chiang AL Galler Rabinowitz L Kumar A Chan WWY: Association between institutional social media involvement and gastroenterology divisional rankings: Cohort study. J Med Internet Res 2019;21:e13345. 31493321 10.2196/13345 PMC6754684

[13] Ciprut S Curnyn C Davuluri M Sternberg K Loeb S: Twitter activity associated with U.S. News and World report reputation scores for urology departments. Urology 2017;108:11-16. 28669746 10.1016/j.urology.2017.05.051

[14] Yousef M Dietrich T Rundle-Thiele S: Social advertising effectiveness in driving action: A study of positive, negative and coactive appeals on social media. Int J Environ Res Public Health 2021;18:5954. 34206119 10.3390/ijerph18115954 PMC8199559

